# The Association between Iron-Deficiency Anemia (IDA) and Septic Arthritis (SA): The Real-World Data

**DOI:** 10.3390/medicina58050617

**Published:** 2022-04-28

**Authors:** Cheng-Hung Chiang, Cheng-Yen Li, Kai-Chieh Hu, Yi-Hsiu Fu, Ching-Chih Chiu, Chia-Chun Hsia, Shu-Jui Kuo, Chih-Hung Hung

**Affiliations:** 1 Department of Orthopedic Surgery, China Medical University Hospital, Taichung 404, Taiwan; rickyjohn0623@gmail.com; 2 Department of Medical Education, Taipei Veterans General Hospital, Taipei 112, Taiwan; daniellee868.dl@gmail.com; 3 Management Office for Health Data, China Medical University Hospital, Taichung 404, Taiwan; billyhu.cmuh@gmail.com; 4 Department of Education, China Medical University Hospital, Taichung 404, Taiwan; yfu1103@gmail.com (Y.-H.F.); kyuuseijimd@gmail.com (C.-C.C.); jimmyhsia0422@gmail.com (C.-C.H.); 5 School of Medicine, China Medical University, Taichung 404, Taiwan

**Keywords:** iron-deficiency anemia, septic arthritis, LHID

## Abstract

*Background and Objectives*: Iron-deficiency anemia (IDA) could predispose the afflicted individuals to various infections and musculoskeletal disorders. This study attempted to investigate the association between IDA and septic arthritis (SA), a musculoskeletal disease. *Materials and Methods*: We investigated all the eligible subjects in the Taiwanese longitudinal health insurance database (LHID) between 2000 and 2012. Subjects with the diagnosis of IDA (International Classification of Diseases, 9th Revision, Clinical Modification (ICD-9-CM): 280) were allocated to the IDA cohort. The control subjects were randomly matched to every subject with IDA coding by age and sex at the 1:4 ratio. All of the recruited subjects were followed since the index date to the onset of SA (ICD-9-CM: 711.0), withdrawal from the insurance (including death), or 31 December 2013. *Results*: The cumulative incidence of SA was assessed. We showed that the cumulative incidence of SA was higher in the IDA cohort than in the control cohort (*p*-value < 0.0001). After adjustment of the comorbidities, the IDA patients had a 2.53-fold risk of SA compared to control subjects (aHR = 2.53, 95% CI = 1.89–3.38). *Conclusions*: IDA was associated with an increased risk of SA.

## 1. Introduction

Iron-deficiency anemia (IDA) is a prevalent disorder affecting more than 2 billion people worldwide [1]. Despite the seemly affable nature, IDA could predispose a person to various infections [1]. For example, IDA was reported to be an independent predictor of respiratory tract infections, and postoperative urinary tract infections were more common in patients with IDA [2]. The susceptibility to various infections among the IDA patients could be partially explained by the compromised humoral and cellular immunity due to iron deficiency [3]. Beyond predisposition to various infections, IDA patients could be susceptible to musculoskeletal disorders. For example, IDA has been proposed as an emerging risk factor for osteoporosis [4]. The impact of IDA on musculoskeletal system is not completely understood at present.

Septic arthritis (SA) is a rapidly progressing and devastating joint disease caused by pathogen infection [5]. The prevalence of SA is around 6 cases per 100,000 in the general population and 70 cases per 100,000 among rheumatoid arthritis (RA) patients [6]. The mortality rate could range from 10–15% for single joint involvement to 30–50% for polyarthritis [5]. The grave prognosis highlighted the importance of identifying the risk factors for SA and being more vigilant for these susceptible subjects.

Despite the fact that IDA patients are more susceptible to infections and that the impact of IDA on musculoskeletal disorders has been implicated, the correlation between IDA and SA, a musculoskeletal infection, is not clear at present. By utilizing the data in the Taiwan National Health Insurance Research Database (NHIRD) for the period 1 January 2000 to 31 December 2012, we examined the association between newly diagnosed IDA and subsequent SA development.

## 2. Materials and Methods

The study used the data derived from the Longitudinal Health Insurance Database (LHID), consisting of the claim data of 1 million beneficiaries randomly selected from the Taiwanese National Health Insurance Research Database (NHIRD). To protect the privacy of the beneficiaries, all of the data were encrypted and anonymized. The claim data comprised of diagnoses of diseases, treatment and prescriptions, and outpatient and inpatient records. The International Classification of Diseases, 9th Revision, Clinical Modification (ICD-9-CM), was used to code and classify the diseases. The study was approved by the Research Ethics Committee at China Medical University and Hospital (CMUH104-REC2-115 (CR4), date of approval: 5 July 2019) [7,8,9].

We identified patients with newly diagnosed IDA (International Classification of Diseases, 9th Revision, Clinical Modification (ICD-9-CM): 280) from LHID concerning both outpatient and inpatient visits. The diagnosis of IDA was defined by the presence of ICD diagnostic coding, and only the subjects with the reimbursement of complete blood-count tests and serum ferritin tests as well as prescription of oral or intravenous iron supplementation were recruited [10].

The index date was defined as the date when the diagnosis of IDA was initially coded. All of the recruited subjects were followed since the index date to the onset of septic arthritis (ICD-9-CM: 711.0), withdrawal from the insurance (including death), or 31 December 2013. The individuals with the diagnosis of SA before the index date, aged less than 20 years, or missing data in the demographic profiles were excluded. The control subjects without IDA coding were randomly matched to every subject with IDA coding by age and sex at the 1:4 ratio.

The comorbidities analyzed in our study included hypertension (ICD-9-CM: 401–405), diabetes mellitus (ICD-9-CM: 250), hyperlipidemia (ICD-9-CM: 272), chronic kidney disease (ICD-9-CM: 585), cancer (ICD-9-CM: 140–208), chronic obstructive pulmonary disease (COPD) (ICD-9-CM: 491, 492, and 496), alcoholic liver disease (ICD-9-CM: 571.0, 571.1, and 571.3), chronic hepatitis (ICD-9-CM: 571.4), hepatitis B (ICD-9-CM: 070.2, 070.3, and V02.61), hepatitis C (ICD-9-CM: 070.41, 070.44, 070.51, 070.54, and V02.62), human immunodeficiency virus (HIV) infection (ICD-9-CM: 042 and V08), and pneumoconiosis (ICD-9-CM: 500–508). The baseline treatments analyzed in our study include splenectomy (ICD-9-CM Procedure Code: 41.5) and gastrectomy (ICD-9-CM Procedure Code: 43.5–43.9). We conducted a sensitivity analysis regarding the association between IDA and susceptibility to various infections. A sensitivity analysis was conducted in the same cohort to explore the association of IDA with infections (ICD-9-CM: 001–041, 045–139, 320–321, 323.0–323.4, 324, 420–421, 422.0, 422.92, 460–466, 475, 478.20–478.24, 480–487, 510, 511.0–511.1, 513, 522.4–522.7, 523.3–523.5, 527.3, 528.3, 569.5, 572.0, 590, 595.89, 595.9, 597.0, 599.0, 601, 604, 614–616) other than SA.

Categorical variables are shown as numbers and percentages. Continuous variables are shown as means and standard deviations (SD). The Student’s *t*-test and the chi-square test were employed to compare continuous and categorical variables, respectively. Incidence rates were presented as the case number per 10,000 person-years. The Kaplan–Meier method was utilized to plot the respective cumulative incidence curves, and the extent of between-curve differences was evaluated by the log-rank test. Both univariable and multivariable versions of Cox’s proportion hazard regression were employed to assess the effect of IDA on the risk of SA, as presented by hazard ratios (HR) with 95% confidence intervals (CI). All analyses were performed employing the SAS statistical software (Version 9.4 for Windows; SAS Institute, Inc., Cary, NC, USA). A two-tailed *p*-value of <0.05 was considered statistically significant.

## 3. Results

Table 1 demonstrates the baseline demographic profiles and comorbidities of the individuals with and without IDA. The composition of gender, age, and HIV infection was homogenous between the two groups. Except HIV infection, the prevalence of the analyzed co-morbidities was significantly higher in the IDA cohort (all *p* < 0.0001 except HIV infection).

**Figure 1:**
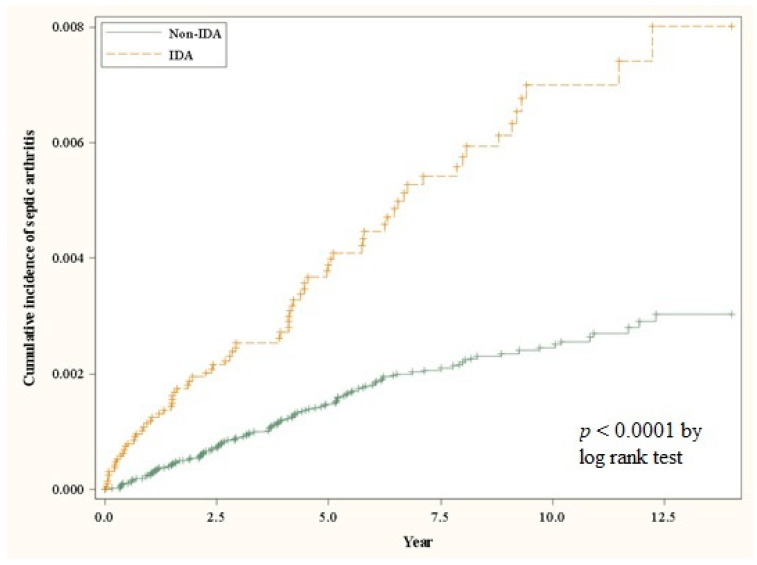
The cumulative incidence curves for the occurrence of septic arthritis for the IDA and control cohorts.

Table 2 demonstrates a multivariate analysis for the confounders that might increase the susceptibility to SA to assess if the IDA is an independent risk factor for SA. According to our model, the incidence of SA in the IDA cohort and in the control cohort was 7.12 and 2.64 per 10,000 person-years, respectively. After adjustment for gender, age, comorbidities, and treatment in baseline, IDA patients had a 2.53-fold risk of SA when compared to the control subjects (aHR: 2.53, 95% CI: 1.89–3.38) (Table 2). The cumulative incidence of SA was significantly higher in the IDA cohort than in the control cohort (log-rank test: *p* < 0.0001) (Figure 1). The factors associated with a higher incidence of SA in our model include advanced age (≥65 years), male gender, hypertension, and chronic kidney disease (Table 2).

Table 3 compares the incidence and adjusted hazard ratio (aHR) of SA between the IDA and the control cohorts stratified by age, gender, comorbidities, and treatment in baseline. The impact of IDA on the occurrence of SA is more prominent (aHR > 2.53) among the subjects with the age of 40–64 years (aHR: 2.89, 95% CI: 1.69–4.97), with male gender (aHR: 3.04, 95% CI: 1.97–4.67), with hypertension (aHR: 2.75, 95% CI: 1.88–4.01), with hyperlipidemia (aHR: 2.79, 95% CI: 1.53–5.09), with COPD (aHR: 2.76, 95% CI: 1.49–5.13), and with chronic hepatitis (aHR: 3.78, 95% CI: 1.76–8.13).

Table 4 compares the incidence and aHR of SA between the IDA and the control cohorts stratified by the duration of follow-up and age. Among the subjects aged between 40–64 years, the impact of IDA was accentuated (aHR > 2.53) when follow-up duration is more than 5 years (aHR: 4.49, 95% CI: 1.94–10.35).

We conducted a sensitivity analysis regarding the association between IDA and infections (ICD-9-CM: 001–041, 045–139, 320–321, 323.0–323.4, 324, 420–421, 422.0, 422.92, 460–466, 475, 478.20–478.24, 480–487, 510, 511.0–511.1, 513, 522.4–522.7, 523.3–523.5, 527.3, 528.3, 569.5, 572.0, 590, 595.89, 595.9, 597.0, 599.0, 601, 604, 614–616) other than SA to elucidate the impact of IDA on general infection sensitivity (Table 5). The incidence of infection in the IDA cohort and in the control cohort was 10.37 and 9.67 per 100 person-years, respectively. After adjustment for gender, age, comorbidities, and treatment in baseline, IDA patients had a 1.07-fold risk of infection when compared to the control subjects (aHR: 1.07, 95% CI: 1.04, 1.09).

## 4. Discussion

Iron is an essential nutrient for all living organisms, including both the hosts and the pathogens. During the acute phase of infection, macrophage iron sequestration deprives the invading pathogens of iron, which leads to growth inhibition. This process, in which the host sequesters key nutrients to reduce pathogenicity, is called nutritional immunity. Anemia of chronic inflammation, however, demonstrates the impact of chronic infection on erythropoietic function due to prolonged macrophage iron sequestration [11]. This implies that IDA not only impairs the physiological function of the hosts but also predisposes them to infection due to the defective capability of eliminating pathogens.

The comprehensive mechanisms for the susceptibility to various infections for IDA patients are not fully understood at present. However, there is substantial evidence inferring the role of iron in human immunity. The innate, humoral, and cellular immunity in the iron-deficient milieu have been investigated in-depth in both humans and animals. In innate immunity, iron modulates the function of phagocytes by regulating enzymes and transcription factors, leading to the production of various microbicidal radicals, including nitric oxide and hydroxyl radical [12,13]. In adaptive immunity, iron has an essential role in cytokine production for lymphocytic clonal expansion. Although the impact of iron deficiency on humoral immunity is inconclusive at present, the impact on cellular immunity is supported by substantial evidence [12,14,15,16,17,18]. As a result, iron deficiency could lead to decreased myeloperoxidase activity in neutrophils, impaired bactericidal activity, decreased T-lymphocyte numbers with thymic atrophy, defective T-lymphocyte-induced proliferative response, impaired natural killer cell activity, impaired lymphocytic interleukin-2 synthesis, and reduced production of macrophage migration inhibition factor [19].

Sickle cell anemia has been shown to be associated with higher incidence of SA among the children, and local vascular insufficiency associated with sickling has been proposed to be the cause. However, as a more common form of anemia, the association between IDA and SA has not been reported before. Despite the known detrimental effects of iron deficiency on the immunity, the correlation between IDA and SA is elusive at present. These indirect but interesting findings in the literature inspired us to investigate the association between IDA and SA.

In our study, we showed that the cumulative incidence of SA was higher in the IDA cohort than in the control cohort (*p*-value < 0.0001). After adjustment, the IDA patients had a 2.53-fold risk of SA compared to control subjects (aHR = 2.53, 95% CI = 1.89–3.38). We also found that the impact of IDA on the occurrence of SA is more prominent among the subjects with the age of 40–64 years, with male gender, with hypertension, with hyperlipidemia, with COPD, and with chronic hepatitis. These results have not been reported before and warrant notice.

There are limitations of our study. There are some subjects with concomitant IDA and anemia of chronic disease. We could only address the problem by excluding the subjects with the coding of anemia of chronic disease, the patients without the reimbursement of serum ferritin tests, and the patients without the prescription of iron supplementation. The microbiologic profiles could not be procured from the NHIRD. The diagnosis of IDA could be underestimated, and the impact of IDA on SA could be skewed towards null hypothesis. However, these limitations could not undermine the strength of our findings concerning the association between IDA and SA.

## 5. Conclusions

This cumulative incidence of SA was higher in the IDA cohort than in the control cohort. The IDA patients had a 2.53-fold risk of SA compared to control subjects. The impact of IDA on the occurrence of SA is more prominent among the subjects with the age of 40–64 years, with male gender, with hypertension, with hyperlipidemia, with COPD, and with chronic hepatitis. The impact of IDA on SA was accentuated among subjects aged between 40–64 years and afflicted with IDA for more than 5 years.

## Figures and Tables

**Table 1:** Demographic characteristics and comorbidities of subjects with and without iron-deficiency anemia (IDA).

Variable	Total	Non-IDA	IDA	*p*-Value
	*N* = 97,390	*N* = 77,912	*N* = 19,478	
	*n*	*n* (%)/Mean ± SD	*n* (%)/Mean ± SD	
Age (year) *				1.0000
20–39	22,015	17,612 (22.60)	4403 (22.60)	
40–64	42,145	33,716 (43.27)	8429 (43.27)	
≥65	33,230	26,584 (34.12)	6646 (34.12)	
Mean ± SD ^a^		55.0 ± 18.6	55.1 ± 18.6	0.6955
Gender *				0.9557
Female	67,336	53,872 (69.14)	13,464 (69.12)	
Male	30,054	24,040 (30.86)	6014 (30.88)	
Comorbidities *				
Hypertension	28,545	21,531 (27.64)	7014 (36.01)	<0.0001
Diabetes mellitus	13,384	9461 (12.14)	3923 (20.14)	<0.0001
Hyperlipidemia	14,054	10,779 (13.83)	3275 (16.81)	<0.0001
CKD	2682	1363 (1.75)	1319 (6.77)	<0.0001
Cancer	2284	1193 (1.53)	1091 (5.60)	<0.0001
COPD	9706	7085 (9.09)	2621 (13.46)	<0.0001
ALD	434	179 (0.23)	255 (1.31)	<0.0001
Chronic hepatitis	7755	5517 (7.08)	2238 (11.49)	<0.0001
Hepatitis B	1984	1400 (1.80)	584 (3.00)	<0.0001
Hepatitis C	1183	745 (0.96)	438 (2.25)	<0.0001
HIV infection	26	17 (0.02)	9 (0.05)	0.0624
Pneumoconiosis	617	389 (0.50)	228 (1.17)	<0.0001
Treatment in baseline *				
Splenectomy	26	8 (0.01)	18 (0.09)	<0.0001
Gastrectomy	116	53 (0.07)	63 (0.32)	<0.0001

ALD, alcoholic liver disease; CKD, chronic kidney disease; COPD, chronic obstructive pulmonary disease; HIV, human immunodeficiency virus; IDA, iron-deficiency anemia; SD, standard deviation. * Chi-square test; ^a^ Student’s *t*-test.

**Table 2:** The incidence rate and hazard ratio of septic arthritis (SA).

Variable	Event	PYs	IR	Crude		Adjusted *	
	*n* = 211		10,000 PYs	HR (95% CI)	*p*-Value	HR (95% CI)	*p*-Value
IDA							
No	133	504,708	2.64	1 (Reference)		1 (Reference)	
Yes	78	109,533	7.12	2.68 (2.03, 3.55)	<0.0001	2.53 (1.89, 3.38)	<0.0001
Age (year)							
20–39	16	156,595	1.02	1 (Reference)		1 (Reference)	
40–64	58	289,851	2.00	1.95 (1.12, 3.39)	0.0180	1.59 (0.91, 2.79)	0.1054
≥65	137	167,794	8.16	7.74 (4.61, 13.01)	<0.0001	4.53 (2.56, 8.04)	<0.0001
Gender							
Female	116	445,865	2.60	1 (Reference)		1 (Reference)	
Male	95	168,376	5.64	2.12 (1.62, 2.78)	<0.0001	1.38 (1.04, 1.83)	0.0258
Comorbidities							
Hypertension	120	149,781	8.01	3.97 (3.02, 5.22)	<0.0001	1.69 (1.22, 2.34)	0.0016
Diabetes mellitus	58	68,024	8.53	2.95 (2.18, 4.00)	<0.0001	1.33 (0.95, 1.86)	0.0923
Hyperlipidemia	47	78,269	6.00	1.92 (1.38, 2.65)	<0.0001	0.94 (0.66, 1.33)	0.7255
Chronic kidney disease	21	10,757	19.52	5.94 (3.78, 9.33)	<0.0001	2.24 (1.40, 3.60)	0.0008
Cancer	6	8846	6.78	1.93 (0.86, 4.34)	0.1134	1.12 (0.49, 2.53)	0.7909
COPD	43	52,632	8.17	2.69 (1.92, 3.76)	<0.0001	1.25 (0.88, 1.78)	0.2054
Alcoholic liver disease	0	2238	0.00	0.00 (0.00, >100)	0.9683	0.00 (0.00, >100)	0.9737
Chronic hepatitis	29	50,483	5.74	1.79 (1.21, 2.65)	0.0036	1.15 (0.76, 1.76)	0.5107
Hepatitis B	8	10,600	7.55	2.18 (1.08, 4.43)	0.0301	1.90 (0.91, 3.95)	0.0855
Hepatitis C	7	5508	12.71	3.64 (1.71, 7.74)	0.0008	1.83 (0.83, 4.04)	0.1355
HIV infection	0	179	0.00	0.00 (0.00, >100)	0.9710	N/A	
Pneumoconiosis	1	2473	4.04	1.16 (0.16, 8.25)	0.8850	0.47 (0.07, 3.36)	0.4502
Treatment in baseline							
Splenectomy	0	64	0.00	0.00 (0.00, >100)	0.9732	N/A	
Gastrectomy	0	524	0.00	0.00 (0.00, >100)	0.9658	NA	

COPD, chronic obstructive pulmonary disease; CI, confidence interval; HIV, human immunodeficiency virus; HR, hazard ratio; IDA, iron-deficiency anemia; IR, incidence rate; N/A, not available; PYs, person-years. * Adjusted for age, sex, comorbidities, and treatment.

**Table 3:** Effects of iron-deficiency anemia (IDA) on septic arthritis (SA) stratified by age, gender, comorbidities, and treatment in baseline.

Variable	Non-IDA			IDA			IDA vs. Non-IDA			
	Event	PYs	IR	Event	PYs	IR	Crude		Adjusted *	
	*n* = 133		10,000 PYs	*n* = 78		10,000 PYs	HR (95% CI)	*p*-Value	HR (95% CI)	*p*-Value
All	133	504,708	2.64	78	109,533	7.12	2.68 (2.03, 3.55)	<0.0001	2.53 (1.89, 3.38)	<0.0001
Age (years)										
20–39	10	125,754	0.80	6	30,841	1.95	2.45 (0.89, 6.74)	0.0828	1.70 (0.57, 5.08)	0.3386
40–64	34	236,559	1.44	24	53,292	4.50	3.14 (1.86, 5.29)	<0.0001	2.89 (1.69, 4.97)	0.0001
≥65	89	142,394	6.25	48	25,400	18.90	2.98 (2.10, 4.24)	<0.0001	2.49 (1.73, 3.58)	<0.0001
Gender										
Female	74	362,679	2.04	42	83,186	5.05	2.47 (1.69, 3.61)	<0.0001	2.20 (1.48, 3.26)	<0.0001
Male	59	142,029	4.15	36	26,347	13.66	3.21 (2.12, 4.86)	<0.0001	3.04 (1.97, 4.67)	<0.0001
Comorbidities										
Hypertension	69	118,975	5.80	51	30,806	16.56	2.84 (1.98, 4.09)	<0.0001	2.75 (1.88, 4.01)	<0.0001
DM	33	51,041	6.47	25	16,983	14.72	2.27 (1.35, 3.82)	0.0020	2.07 (1.21, 3.54)	0.0081
Hyperlipidemia	26	61,864	4.20	21	16,404	12.80	3.06 (1.72, 5.44)	0.0001	2.79 (1.53, 5.09)	0.0008
CKD	10	5477	18.26	11	5280	20.83	1.18 (0.50, 2.78)	0.7048	1.39 (0.58, 3.35)	0.4641
Cancer	2	5413	3.69	4	3433	11.65	3.24 (0.59, 17.69)	0.1751	2.67 (0.35, 20.14)	0.3398
COPD	24	41,047	5.85	19	11,585	16.40	2.76 (1.51, 5.04)	0.0010	2.76 (1.49, 5.13)	0.0013
ALD	0	1166	0.00	0	1072	0.00	N/A		NA	
Chronic hepatitis	12	37,694	3.18	17	12,789	13.29	4.11 (1.96, 8.61)	0.0002	3.78 (1.76, 8.13)	0.0007
Hepatitis B	4	7885	5.07	4	2715	14.74	2.98 (0.74, 11.91)	0.1229	2.44 (0.58, 10.37)	0.2261
Hepatitis C	3	3912	7.67	4	1596	25.06	3.21 (0.72, 14.40)	0.1280	3.95 (0.84, 18.62)	0.0829
HIV infection	0	130	0.00	0	49	0.00	N/A		N/A	
Pneumoconiosis	1	1857	5.39	0	617	0.00	N/A		N/A	
Baseline treatment										
Splenectomy	0	29	0.00	0	35	0.00	N/A		N/A	
Gastrectomy	0	278	0.00	0	247	0.00	N/A		N/A	

ALD, alcoholic liver disease; COPD, chronic obstructive pulmonary disease; CI, confidence interval; CKD, chronic kidney disease; DM, diabetes mellitus;. HIV, human immunodeficiency virus; HR, hazard ratio; IDA, iron-deficiency anemia; IR, incidence rate; N/A, not available; PYs, person years. * Adjusted for age, sex, comorbidities, and treatment.

**Table 4:** The incidence of septic arthritis stratified by age and follow-up periods.

Variable	Non-IDA			IDA			IDA vs. Non-IDA			
	Event	PYs	IR	Event	PYs	IR	Crude		Adjusted *	
			10,000 PYs			10,000 PYs	HR (95% CI)	*p*-Value	HR (95% CI)	*p*-Value
Age < 40 years										
<2	1	2630	3.80	4	717	55.77	15.11 (1.69, >100)	0.0152	11.61 (0.93, >100)	0.0569
2–5	6	15,859	3.78	1	3920	2.55	0.68 (0.08, 5.68)	0.7258	0.55 (0.06, 5.46)	0.6084
≥5	3	107,265	0.28	1	26,203	0.38	1.37 (0.14, 13.14)	0.7864	1.11 (0.11, 10.72)	0.9311
Age 40–64 years										
<2	6	4806	12.48	6	1566	38.32	3.10 (1.00, 9.65)	0.0504	2.43 (0.73, 8.02)	0.1464
2–5	16	31,625	5.06	7	7907	8.85	1.79 (0.74, 4.35)	0.1998	1.65 (0.66, 4.15)	0.2843
≥5	12	200,128	0.60	11	43,819	2.51	4.23 (1.87, 9.59)	0.0006	4.49 (1.94, 10.35)	0.0004
Age ≥ 65 years										
<2	33	6472	50.99	24	2254	106.50	2.22 (1.31, 3.77)	0.0030	2.19 (1.27, 3.77)	0.0047
2–5	31	30,527	10.16	14	6876	20.36	2.10 (1.12, 3.95)	0.0214	1.67 (0.87, 3.22)	0.1244
≥5	25	105,396	2.37	10	16,270	6.15	2.71 (1.30, 5.65)	0.0076	2.25 (1.05, 4.82)	0.0360

CI, confidence interval; HR, hazard ratio; IDA, iron-deficiency anemia; IR, incidence rate; PYs, person years. * Adjusted for age, sex, comorbidities, and treatment.

**Table 5:** The sensitivity of IDA patients to infections other than SA.

Variable	Event	Person-Year	IR	Crude		Adjusted *	
	*n* = 44,434		100 Person-Years	HR (95% CI)	*p*-Value	HR (95% CI)	*p*-Value
**IDA**							
No	35,589	367,934	9.67	1 (Reference)		1 (Reference)	
Yes	8845	85,266	10.37	1.08 (1.06, 1.11)	<0.0001	1.07 (1.04, 1.09)	<0.0001
**Age (year)**							
20–39	10,198	109,205	9.34	1 (Reference)		1 (Reference)	
40–64	19,513	204,647	9.53	1.03 (1.01, 1.06)	0.0078	1.02 (1.00, 1.05)	0.0608
≥65	14,723	139,347	10.57	1.21 (1.18, 1.24)	<0.0001	1.16 (1.13, 1.20)	<0.0001
**Gender**							
Female	31,134	321,502	9.68	1 (Reference)		1 (Reference)	
Male	13,300	131,698	10.10	1.08 (1.06, 1.10)	<0.0001	1.02 (1.00, 1.05)	0.0299
**Comorbidities**							
Hypertension	12,286	120,354	10.21	1.11 (1.09, 1.14)	<0.0001	1.01 (0.99, 1.04)	0.3027
Diabetes mellitus	5746	55,024	10.44	1.12 (1.09, 1.16)	<0.0001	1.06 (1.03, 1.10)	<0.0001
Hyperlipidemia	5739	59,955	9.57	1.01 (0.99, 1.04)	0.3322	0.94 (0.91, 0.97)	<0.0001
Chronic kidney disease	1094	9873	11.08	1.19 (1.12, 1.26)	<0.0001	1.08 (1.02, 1.15)	0.0138
Cancer	978	8476	11.54	1.23 (1.15, 1.31)	<0.0001	1.15 (1.08, 1.23)	<0.0001
COPD	4766	43,088	11.06	1.15 (1.12, 1.19)	<0.0001	1.06 (1.03, 1.10)	0.0001
Alcoholic liver disease	181	1789	10.12	1.05 (0.91, 1.21)	0.5173	1.01 (0.87, 1.17)	0.8593
Chronic hepatitis	3838	37,286	10.29	1.05 (1.01, 1.08)	0.0055	1.02 (0.99, 1.06)	0.2234
Hepatitis B	793	8223	9.64	1.03 (0.96, 1.11)	0.3799	1.03 (0.95, 1.10)	0.4897
Hepatitis C	450	4597	9.79	1.05 (0.96, 1.15)	0.2963	0.98 (0.89, 1.08)	0.6408
HIV infection	10	122	8.18	0.84 (0.46, 1.56)	0.5905	0.83 (0.45, 1.55)	0.5683
Pneumoconiosis	301	2278	13.21	1.37 (1.23, 1.54)	<0.0001	1.21 (1.08, 1.36)	0.0008
**Treatment in baseline**							
Splenectomy	11	96	11.46	1.18 (0.65, 2.12)	0.5885	0.95 (0.52, 1.74)	0.8648
Gastrectomy	51	477	10.70	1.12 (0.85, 1.48)	0.4103	0.94 (0.71, 1.25)	0.6903

CI, confidence interval; HR, hazard ratio; IDA, iron-deficiency anemia; IR, incidence rate; PYs, person years. * Adjusted for age, sex, comorbidities, and treatment.

## Data Availability

The data presented in this study are available on request from the corresponding author.

## References

[1] Camaschella C. (2015). Iron-deficiency anemia. N. Engl. J. Med..

[2] Tansarli G.S., Karageorgopoulos D.E., Kapaskelis A., Gkegkes I., Falagas M.E. (2013). Iron deficiency and susceptibility to infections: Evaluation of the clinical evidence. Eur. J. Clin. Microbiol. Infect. Dis..

[3] Jonker F.A.M., Te Poel E., Bates I., Boele van Hensbroek M. (2017). Anaemia, iron deficiency and susceptibility to infection in children in sub-Saharan Africa, guideline dilemmas. Br. J. Haematol..

[4] Toxqui L., Vaquero M.P. (2015). Chronic iron deficiency as an emerging risk factor for osteoporosis: A hypothesis. Nutrients.

[5] Jin T., Mohammad M., Pullerits R., Ali A. (2021). Bacteria and Host Interplay in Staphylococcus aureus Septic Arthritis and Sepsis. Pathogens.

[6] Tarkowski A. (2006). Infection and musculoskeletal conditions: Infectious arthritis. Best Pr. Res. Clin. Rheumatol..

[7] Lai S.W., Liao K.F., Lin C.L., Lin C.H. (2020). Association between Parkinson’s disease and proton pump inhibitors therapy in older people. Biomedicine.

[8] Lai S.W., Liao K.F., Lin C.L., Lin C.C., Lin C.H. (2020). Longitudinal data of multimorbidity and polypharmacy in older adults in Taiwan from 2000 to 2013. Biomedicine.

[9] Lai S.W., Lin C.L., Liao K.F. (2020). Hyperuricemia might be an early manifestation of undiagnosed adult leukemia in a population-based cohort study. Biomedicine.

[10] Chu K.A., Hsu C.H., Lin M.C., Chu Y.H., Hung Y.M., Wei J.C. (2019). Association of iron deficiency anemia with tuberculosis in Taiwan: A nationwide population-based study. PLoS ONE.

[11] Nairz M., Weiss G. (2020). Iron in infection and immunity. Mol. Aspects Med..

[12] Cronin S.J.F., Woolf C.J., Weiss G., Penninger J.M. (2019). The Role of Iron Regulation in Immunometabolism and Immune-Related Disease. Front. Mol. Biosci..

[13] Nairz M., Theurl I., Swirski F.K., Weiss G. (2017). “Pumping iron”-how macrophages handle iron at the systemic, microenvironmental, and cellular levels. Pflug. Arch..

[14] Cronin S.J.F., Seehus C., Weidinger A., Talbot S., Reissig S., Seifert M., Pierson Y., McNeill E., Longhi M.S., Turnes B.L. (2018). The metabolite BH4 controls T cell proliferation in autoimmunity and cancer. Nature.

[15] Batista A., Millan J., Mittelbrunn M., Sanchez-Madrid F., Alonso M.A. (2004). Recruitment of transferrin receptor to immunological synapse in response to TCR engagement. J. Immunol..

[16] Vanoaica L., Richman L., Jaworski M., Darshan D., Luther S.A., Kuhn L.C. (2014). Conditional deletion of ferritin h in mice reduces B and T lymphocyte populations. PLoS ONE.

[17] Jiang Y., Li C., Wu Q., An P., Huang L., Wang J., Chen C., Chen X., Zhang F., Ma L. (2019). Iron-dependent histone 3 lysine 9 demethylation controls B cell proliferation and humoral immune responses. Nat. Commun..

[18] Minton K. (2008). Ironing out the causes of B-cell dysfunction. Nat. Rev. Immunol..

[19] Kumar V., Choudhry V.P. (2010). Iron deficiency and infection. Indian J. Pediatr..

